# Supplementary material for: Unique dental arrangement in a new species, Groenlandaspis howittensis (Placodermi, Arthrodira) from the Middle Devonian of Mount Howitt, Victoria, Australia
Source: PeerJ. 2024 Dec 23;12:e18759. doi: 10.7717/peerj.18759 (PMC11670761; doi:10.7717/peerj.18759)
Supplement: Supplemental Information 3 [file peerj-12-18759-s003.docx]

Modifications from Zhu *et al.* (2016) phylogenetic matrix

*Dicksonosteus arcticus*

( 1 ) 0 > 1

(51) 0 > 1

(63 ) ? > 1

(77) 1 > 0

*Groenlandaspis antarcticus*

(24) 1 > 0

(26) 0 > -

(30) 0 > 1

(51) ? > 1

(53) ? > 0

(77) 1 > 0

*Turrisaspis elektor*

(13) 0 > 1

(20) 0 > 1

(26) 0 > -

(50) ? > 0

(53) ? > 0

(77) 1 > 0

*Elvaspis tuberculata*

(51) 0 -> 1

*Kujdowniaspis podolica*

(11) 0 > 2

(31) 1 > 0

Zhu Y-A, Zhu M, Wang, J-Q. 2016. Redescription of Yinostius major (Arthrodira: Heterostiidae) from the Lower Devonian of China, and the interrelationships of Brachythoraci. *Zoological Journal of the Linnean Society* 176(4):806-34. [DOI doi.org/10.1111/zoj.12356](https://doi.org/10.1111/zoj.12356)
