# Supplementary material for: Unique dental arrangement in a new species, Groenlandaspis howittensis (Placodermi, Arthrodira) from the Middle Devonian of Mount Howitt, Victoria, Australia
Source: PeerJ. 2024 Dec 23;12:e18759. doi: 10.7717/peerj.18759 (PMC11670761; doi:10.7717/peerj.18759)
Supplement: Supplemental Information 4 [file peerj-12-18759-s004.docx]

**1 – 85 Carr (1991)**

# Nuchal plate with posterodorsally placed paired pits (f.lv of Goujet, 1984): absent (0), present on the paranuchal plate in *Holonema*; present (1).

# Posterolateral extension of the lateral consolidated arch: extends as a distinct ridge or thickening onto postmarginal plate, or to the skull roof edge if the postmarginal plate is absent (0); extends onto the marginal plate (1); absent (2).

# Triangular depression posterior to supraorbital vault (character 14, in part, of Dennis & Miles, 1979b): absent (0); present (1).

# Neurocranial process or thickening (character 16, in part, of Dennis & Miles, 1979b): absent (0); thickening (1); distinct process (2).

# Rostral plate posterior development: not developed (0); subtriangular (1); T-shaped (2).

# Anterior elongation of the rostrum (Dennis & Miles, 1979b: fig. 18, character 3, in part; Long, 1988: characters 4, 12, and 16): absent (0); present (1).

# Ventrally enclosed tubular rostrum: absent (0); present (1).

# Position of anterior edge of pineal plate: over orbit (0); anterior to orbit (1).

# Position of posterior edge of pineal plate: over orbit (0); posterior to orbit (1).

# Shape of the external anterior nuchal border: straight (0); convex (1); concave (2).

# Shape of the posterior nuchal border: straight (0); convex (1); concave (2).

# Nuchal thickening (Lelièvre, 1988): discontinuous with the lateral thickening associated with the craniothoracic dermal articulation (0); continuous thickening across the posterior skull roof (1).

# Preorbital plate embayment of the central plate: absent (0); shallow (1); developed (2).

# Paranuchal plate embayment of the central plate: absent (0); shallow, determined by formation of the lateral lobe, and not the posterior lobe (1); developed (2).

# Ratio for the external longitudinal lengths of the preorbital and central plates (r = PrO/C): subequal, i.e. 0.75 < r < 1.25 (0); preorbital plate longer, i.e. r > 1.25 (1); central plate longer, i.e. r < 0.75 (2).

# Postnasal plate infrafenestral process (Gardiner & Miles, 1990: character 22.18): absent (0); present (1).

# Preorbital plate with a dermal preorbital lamina or process (Dennis & Miles, 1979b: character 19): absent (0); present (1).

# Preorbital plates with a median contact (Young, 1981: modified after character 19a): contact posterior to pineal (0); contact anterior to pineal (1); pineal separates preorbital plates (2).

# Extent of median preorbital plate contact: short (0); long, measured as the ratio of (contact length)/(PrO parasagittal length) (1).

# Postorbital plate embayment of the central plate: absent (0); shallow (1); developed (2).

# Length of central plate contact with the nuchal plate: short (0); long, measured as greater than one half the length from the nuchal anteromedian point to the posterolateral corner of the nuchal plate (1).

# Width of the central plate at its contact with the pineal plate: central plate tapers anteriorly to contact (0); central plate expanded laterally to produce a transverse anterior border (1).

# Marginal plate in contact with the central plate (character 5 of Lelièvre, 1988): absent (0); present (1).

# Extent of marginal plate along the lateral border of the skull roof: less than halfthe length from the postorbital process to the posterolateral corner of the skull roof (0); greater than half the length (1).

# Marginal plate forms part of orbit border (Dennis & Miles, 1979b: character 26): absent (0); present (1).

# Postnasal plate forms part of the orbit border (Dennis & Miles, 1979b: fig. 18, character 1): absent (0); present (1).

# Extent of postnasal involvement in the orbit border: minimal (0); developed (1).

# Orbit size: measured as a ratio of (distance between preorbital and postorbital dermal processes)/(distance between pineal and paired condyles): small (0); intermediate (1); large (2).

# Nuchal and paranuchal plates elongate (Lelièvre, 1984b): absent (0); present, associated with an elongation of the endolymphatic canal (1).

# Postnuchal process of paranuchal plate on dermal surface (Dennis & Miles, 1979b: modified after character 8): absent (0); present (1), indicated by the presence of a postnuchal process on the dermal surface of the skull roof.

# Position of the central/paranuchal/marginal or postorbital triple point (plate junction) relative to the paranuchal/central/nuchal triple point: C/PNu/M or PtO triple point anterior to PNu/ C/Nu (0); posterior to PNu/C/Nu (1).

# Position of preorbital/postorbital/central triple point relative to posterior triple point (PtO/M/C or PtO/PNu/C): parallels the midline or with the anterior point lateral (0); posterior point lateral to anterior point (1).

# Position of the PrO/PtO/C triple point relative to the orbit: posterior to orbit (0); over the orbit (1).

# Postorbital dermal process; negligible or absent (0); present (1).

# Median dorsal plate with a ventral ridge (Dennis & Miles, 1979a: combination of characters 4 and 18): a ventral thickening or ridge (0); posterior ridge with a posterior process, not spatulate (1); posterior process posterodorsally spatulate (2).

# Median dorsal plate shape (width/length): short and broad to equidimensional (0); body of plate long and narrow, not including a posteriorly produced spine (1).

# Median dorsal plate posterior border (Dennis & Miles, 1979a: character 18): rounded (0); spinous (1).

# Anterior lateral plate/anterior ventrolateral plate contact: absent (0); present (1).

# Anterior lateral plate/interolateral plate external contact: absent (0); present (1).

# Interolateral/spinal lateral contact: absent (0); present (1).

# Pectoral dermal enclosure: fenestra (0); embayment opens posteriorly (1).

# Posterior lateral plate and posterior dorsolateral plate overlap (Vézina, 1988): simple overlap (0); insertion (1).

# Posterior lateral plate forms part of closed pectoral fenestra: absent (0); present (1).

# Posterior lateral plate ventral lamina (Gardiner & Miles, 1990: character 22.13): absent (0); present (1).

# Spinal plate (Stensiö, 1959): absent (0); present (1).

# Spinal plate spinal pit (Gardiner & Miles, 1990: character 22.32): absent (0); present (1).

# Interolateral plate branchial lamina: absent (0); present (1).

# Interolateral plate ventral lamina: small or absent (0); developed with either an enlarged contact with the anterior median ventral plate, or an enlarged overlap with the anterior ventrolateral plate (1).

# Interolateral plate branchial lamina ornamentation: absent (0); present (1).

# Posterior ventrolateral plate dorsolateral contact with the lateral thoracic armour: posterior lateral plate only (0); posterior lateral and anterior lateral plates (1).

# Posterior ventrolateral plate postpectoral lamina (Gardiner & Miles, 1990: character 22.8): absent (0); small (1); large (2).

# Suborbital dermal lamina (Dennis & Miles, 1979a: character 17): absent (0); present (1).

# Suborbital plate overlaps postorbital plate (Gardiner & Miles, 1990: character 22.9): absent (0); present (1).

# Suborbital/preorbital contact: absent (0); lateral contact (1).

# Suborbital and marginal plates in contact (Gardiner & Miles, 1990: character 22.28): absent (0); present (1).

# Suborbital plate with a contact face for the PSG (cf.PSG) on the linguiform process (cr.lg): absent (0); present (1).

# Palatoquadrate ossification (Dennis & Miles, 1979a: character 21): ossified as a single unit (0); autopalatine and quadrate ossifications (1).

# Detent process on quadrate (Gardiner & Miles, 1990: character 22.1): absent (0); present (1).

# Submarginal plate closely associated with the hyomandibular: absent (0); present, indicated by the presence of perichondral bone or the presence of an internal depression (1).

# Form of the submarginal plate: broad (0); elongate (1).

# Length of cheek and skull roof contact: long (0); short, measured as the contact being less than one third of the length measured from the anterior suborbital plate to the posterior cheek/skull roof contact (1).

# Development of cheek/skull roof overlap: loose contact (0); well bound contact, indicated by the interdigitation of plates or their complete fusion (1).

# Inferognathal with a developed blade portion (Dennis & Miles, 1979a: modified after character 13); absent (0); present (1).

# Inferognathal anterior dental field developed anteriorly of the cusp region: absent (0); present (1), indicated by a projection of the inferognathal anterior to the dorsal functional region.

# Adsymphyseal (anterior dental field) denticles: absent (0); present (1).

# Anterior superognathal cusps: none (0); lateral (1); lateral and anterior (2).

# Anterior superognathal plate with an enclosed lateral face: absent (0); present (1).

# Anterior superognathal plate with a dorsal process (Dennis & Miles, 1979a: character 22, in part): absent (0); present (1).

# Dorsal process of the anterior superognathal plate forms an open ring (Lelièvre et al., 1981: modified after character 4): ring open, measured as greater than 90 ° (0); ring forms a ~90 ° angle (1); ring closed, measured angle less than 90 ° (2).

# Posterior superognathal plate with a dorsal process (Dennis & Miles, 1979a: character 22, in part): absent (0); large (1); small (2).

# Width/length ratio for the parasphenoid pre- and posthypophysial shelf: prehypophysial shelf width greater than length, posthypophysial shelf width greater than length (0); prehypophysial shelf width less than length, posthypophysial shelf width greater than length (1); prehypophysial shelf width greater than length, posthypophysial shelf width less than length (2); prehypophysial shelf width less than length, posthypophysial shelf width less than length (3).

# Parasphenoid posterolateral process (Gardiner & Miles, 1990: character 22.36): absent (0); present (1).

# Median hypophysial vein foramen (Gardiner & Miles, 1990: character 22.7): absent (0); present (1).

# Parasphenoid ventromedian crest (Gardiner & Miles, 1990: character 22.27): absent (0); present (1).

# Angle between the postorbital and otic branches of the infraorbital sensory line groove (modified after character 5 of Lelièvre et al., 1987): open posteriorly (i.e. the formed angle is greater than 90 °) (0); partially closed (i.e. the formed angle is between 45 ° and 90 °) (1); closed (i.e. the formed angle is less than 45 °) (2).

# Central canal groove meets supraorbital groove: absent (0); present (1).

# Ventral groove of the main lateral line on the anterior dorsolateral plate (lc.vl of Miles, 1971: fig. 108): absent (0); present (1).

# Dorsal groove of the main lateral line on the posterior dorsolateral plate (Miles, 1971: ld, fig. 108): absent (0); present (1), distinguished from the main lateral line groove by its dorsal trajectory or its continuation onto the median dorsal plate.

# Main lateral line groove on the posterior dorsolateral plate (lc of Miles, 1971, fig. 108): absent (0); present (1).

# Dorsal groove of the main lateral line on the median dorsal plate (Miles, 1971: ld, fig. 108): absent (0); present (1).

# Postorbital sensory line groove on suborbital plate (Lelièvre, 1988: character 1): absent (0); present (1).

# Angle between the suborbital and postorbital branches of the infraorbital sensory line groove (Lelièvre, 1984a): opened (0); closed, measured as less than 90 ° (1).

# Postorbital branch of the infraorbital sensory line groove and supraoral sensory groove confluent (Lelièvre, 1984a): discontinuous (0); continuous (1).

# Supraorbital canal extended onto central plates: absent (0); present (1).

# Postmarginal canal: absent (0); present (1).

**86 – 98, Carr 2010; characters 92, 93 modified from Zhu & Zhu 2013**

# Anterior superognathal articulation with parasphenoid (Dunkle & Bungart, 1946): absent (0); present, indicated by either articular facets or a thickened contact face (1).

# Parasphenoid anterior contact: thickened anterolateral contact face (0); transverse articular facets (1). In taxa lacking a parasphenoid–gnathal contact, this character is scored as ‘not applicable’.

# Lateral face of anterior superognathal with an occlusal shelf posterior to the lateral cusp: absent (0); present (1).

# Inferognathal with a secondary thickened ridge in the occlusal region, which may be associated with a secondary cusp: absent (0); present (1).

# Skull roof and thoracic armour articulation (Goujet, 1984): articulation medial (0); articulation laterally displaced (1).

# Dermal ornamentation: absent (0); present (1).

# Position of the posterolateral corner of the skull roof judged from the ratio (r1) of the posterior sensory line junction–fossa distance (distance c) divided by the anterior sensory line junction–fossa distance (distance b), r1 < 0.45 (0), 0.45 = < r1 = < 0.58 (1), r1 > 0.58, (2)

# Extension of the anterior skull roof judged from the ratio (r2) of the anterior sensory line junction–pineal foramen distance (distance d) divided by the anterior sensory line junction–fossa distance (distance b), r2 < 0.3 (0), 0.3 = < r2 = < 0.5 (1), r2 > 0.5, (2).

# In forms with a marginal–central plate contact (e.g. Fig. 8), position of the junction of the postorbital, marginal, and central plates, relative to the anterior margin ofthe nuchal plate (modified from Long, 1987, character 7): anterior (0); posterior (1). In taxa lacking a marginal–central plate contact, this character is scored as ‘not applicable’.

# In forms with a marginal–central plate contact (e.g. Fig. 8), position of the junction of the marginal, paranuchal, and central plates, relative to the anterior margin of the nuchal plate (modified from Long, 1987, character 7): anterior (0); posterior (1). In taxa lacking a marginal–central plate contact, this character is scored as ‘not applicable’.

# In forms without a marginal–central plate contact, position of the junction between the postorbital, paranuchal, and central plates, relative to the anterior margin of the nuchal plate (modified from Long, 1987, character 7): anterior (0); posterior (1). In taxa possessing a marginal– central plate contact, this character is scored as ‘not applicable’.

# Paired pits on the internal side of the nuchal plate (pt.u; Fig. 6A) separated by a median septum (m.sept; Fig. 6A) that is continuous with the median posterior process (p.pr; Fig. 6A, C): absent (0); present (1).

# Presence of a supraorbital vault intimately associated with the lateral consolidated arch (Carr, 1991): absent (0); present (1).

**Characters 99 – 121, Zhu *et al.* 2015**

# Development of the crista supraethmoidalis at the visceral surface of the skull roof: underdeveloped, absent or present but not convergent in the midline (0); developed, horizontally across the visceral surface of the skull roof (1).

# Rostral and pineal plates fused into one plate: absent (0); present (1).

# Shape of the pineal plate on the external surface of the skull roof: short and broad (0); long and narrow (1).

# Lateral development of the supraorbital vault compared with the breadth of the skull roof at the level immediately behind the supraorbital vault. This definition of the skull roof breadth is to make this character independent to character 113: underdeveloped, indicated by the ratio of the breadth of both sides of the lateral expansion of the supraorbital vaults/the breadth of the skull roof at the level immediately behind the supraorbital vault less than 0.3 (0); developed, indicated by the ratio of the breadth of the lateral expansion of the supraorbital vaults/the breadth of the skull roof larger than 0.3 (1).

# Development of the postorbital process of the postorbital plate: moderately developed (0); highly developed, defined as the postorbital process composing one-quarter of the orbit (1).

# Position of the anterior end of the endolymphatic duct on the visceral surface of the skull roof, indicating the neurocranial aperture of the endolymphatic duct: anteriorly positioned, defined as positioned anterior to the nuchal thickening (0); posteriorly positioned, on the nuchal thickening (1).

# Posterior lateral shape of the nuchal plate: nuchal straight, not expanded laterally (0); nuchal trapezoid, expanded laterally (1).

# Median ridge on the visceral surface of the nuchal plate, corresponding to the median depression on the occipital region of the neurocranium: absent (0); present (1).

# Position of the paired nuchal pits: anteriorly positioned, defined by position anterior to or on the transverse nuchal thickening (0); posteriorly positioned, defined by position posterior to the transverse nuchal thickening (1).

# Position of the skull roof and thoracic armour articulation: not close to posterolateral corner of the skull roof (0); close to posterolateral corner of the skull roof (1).

# Skull roof fenestra: absent (0); present (1).

# Shape of the infragnathal plate, defined by the depth of the occlusal portion and posterior blade portion, respectively: blade portion significantly deeper (0); occlusal portion deeper, or nearly equal between these two parts of the infragnathal plate (1). Anderson (2008) identified two distinct morphotypes in eubrachythoracids indicating different feeding niches. The deep blade and narrow dental portion in most coccosteomorphs implies a scissor-like occlusion, while the narrow blade and deep dental portion in most pachyosteomorphs implies a vice-like occlusion.

# Parasphenoid thickened around the buccohypophysial foramen: absent (0); present (1).

# Stem-like prehypophysial region of the parasphenoid: absent (0); present (1).

# Breadth of the orbitotemporal neurocranium, indicated by the breadth between the two inner margins of the supraorbital vaults: wide, defined by the ratio of the breadth of the obitotemporal neurocranium/the breadth of the skull roof at the level immediately behind the supraorbital vault larger than 0.4 (0); narrow, defined by the ratio of the breadth of the obitotemporal neurocranium/ the breadth of the skull roof less than 0.4 (1).

# Shape of the supravagal process of the neurocranium, indicated by the impression of the channel left by the supravagal process on the visceral surface of the skull roof when the neurocranium is not preserved: near rightangled (0); developed laterally to form an acute angle (1).

# Posterior development of the posterior carinal process of the keel on the visceral surface of the median dorsal plate: posteriorly developed, beyond the posterior margin of the median dorsal plate in dorsal view (0); not posteriorly developed, not beyond the posterior margin of the median dorsal plate in dorsal view (1).

# Median dorsal plate elevated dorsally into a median crest: (0) absent; present (1).

# Anterior dorsolateral plate and anterior lateral plate fused into one complex: not fused (0); fused (1).

# Anterior ventral corner or anterior ventral wing (sensu Carr, 1996) of the anterior lateral plate extends anterolaterally: not extending anterolaterally, the lateral profile of the anterior lateral plate is sub-triangular or rhomboid (0); extending anterolaterally, making the lateral profile of the anterior lateral plate ‘boomerang’-shaped (1).

# In those taxa that possess an anterolaterally extended anterior lateral plate, the extent of the extension: extended normally (0); extended into a rod-like structure (1). In those taxa that do not possess an anterolaterally extended anterior lateral plate, this character is scored as ‘not applicable’.

# External surface of the anterior lateral plate quadrated by four ridges radiating from the ossification center of the plate: absent (0); present (1).

# Anteroventral groove on the ventral surface of the interolateral plate: absent (0); present (1).

**Characters 122 – 133, this article.**

# Cervical Joint: Sliding (0); Ginglymoid (1).

# Transversely divided pineal plate forming anterior and posterior plates: Absent (0); Present (1).

# Cutaneous sensory pits present on the suborbital or/and post suborbital plates (King et al. 2018): Absent (0); Present (1).

# Dermal contact between the anterior dorsolateral and posterior lateral plates: Absent (0); Present (1).

# Inverted V-shaped flexure of the posterior dorsolateral plate sensory canal. Scored not applicable in taxa without a PDL sensory canal (Long 1995): No flexure (0) Weak flexure, >110° (1) Strong flexure, <110° (2).

# Dorsolateral ridge originating from near the condyle of the anterior dorsolateral plate (Long 1995): Absent (0) Present (1).

# Medial contact of the dorsolateral plates under the median dorsal plate (Goujet 1984): No contact (0); Anterior dorsolateral plates (1); Anterior and posterior dorsolateral plates (2).

# Internal annular thickening of the posterior trunk plates (‘b.cpd’, Goujet 1984, fig. 61B): Absent (0); Present (1).

# Median contact of the posterior ventrolateral plate (Goujet 1984, Dupret 2004: Simple overlap (0); Sigmoidal/double overlapping (1)

# Ventral sensory canals: Absent (0) Present (1)

# Distinct infraspinal lamina/process (‘pr.infsp’, Miles & Westoll 1968, fig. 40C; ‘la.spv’, Goujet 1984, fig. 66A) of the anterior ventrolateral plate: Absent (0) Present (1).

# Anterior ventral plates: Absent (0) Present (1)

**References**

Anderson, P. S. L. (2008). Shape Variation Between Arthrodire Morphotypes Indicates Possible Feeding Niches*. Journal of vertebrate paleontology*, 28(4), 961-969. DOI 10.1671/0272-4634-28.4.961

Carr, RK. 1991. Reanalysis of *Heintzichthys gouldii* (Newberry), an aspinothoracid arthrodire (Placodermi) from the Famennian of northern Ohio, with a review of brachythoracid systematics. *Zoological Journal of the Linnean Society*, 103(4): 349-390.

Carr, RK. 1996. *Stenosteus Augustopectus* sp. nov. from the Cleveland Shale (Famennian) of Northern Ohio with a Review of Selenosteid (Placodermi) Systematics. *Kirtlandia*, 49: 19-43.

Carr RK, Hlavin WJ. 2010. Two new species of *Dunkleosteus* Lehman, 1956, from the Ohio Shale Formation (USA, Famennian) and the Kettle Point Formation (Canada, Upper Devonian), and a cladistic analysis of the Eubrachythoraci (Placodermi, Arthrodira). *Zoological Journal of the Linnean Society*, 159(1): 195-222. [DOI 10.1111/j.1096-3642.2009.00578.x](https://doi.org/10.1111/j.1096-3642.2009.00578.x)

Dennis KD, Miles RS. 1979a. A second eubrachythoracid arthrodire from Gogo, Western Australia. *Zoological Journal of the Linnean Society* 67: 1–29.

Dennis KD, Miles RS. 1979b. Eubrachythoracid arthrodires with tubular rostral plates from Gogo, Western Australia. *Zoological Journal of the Linnean Society* 67: 297–328.

Dunkle DH, Bungart PA. 1946. The antero-supragnathal of Gorgonichthys. *American Museum Novitates* 1316: 1–10.

Dupret, V. (2004). The phylogenetic relationships between actinolepids (Placodermi: Arthrodira) and other arthrodires (phlyctaeniids and brachythoracids). Lower vertebrates from the Palaeozoic: First International Palaeontological Congress Sydney, Australia, July 2002,

Gardiner BG, Miles RS. 1990. A new genus of eubrachythoracid arthrodire form Gogo, Western Australia. *Zoological Journal of the Linnean Society* 90: 159–204.

Goujet D. 1984. Les poissons placodermes du Spitsberg—Arthrodires Dolichothoraci de la 626 Formation de Wood Bay (Devonien inferieur), Cahiers de Paleontologie (section 627 vertebres), Editions du CNRS, Paris.

King B, Hu Y, Long, JA. 2018. 'Electroreception in early vertebrates: survey, evidence and new information. *Palaeontology*, 61(3): 325-58. [DOI 10.1111/pala.12346](https://doi.org/10.1111/pala.12346)

Lelièvre H. 1984a. *Atlantidosteus hollardi* n. g., n. sp., nouveau Brachythoraci (Vertébrés, Placodermes) du Dévonien inférieur du Maroc présaharien. *Bulletin du Museum national d’Histoire naturelle, Paris* 4: 197–208.

Lelièvre H. 1984b. *Antineosteus lehmani* n. g., n. sp., nouveau Brachythoraci du Dévonien inférieur du Maroc présaharien. *Annales de Paléontologie* 70: 115–158.

Lelièvre H. 1988. Nouveau matériel d’*Antineosteus lehmani* Lelièvre, 1984 (Placoderme, Brachythoraci) et d’Acanthodiens du Dévonien inférieur (Emsien) d’Algérie. *Bulletin du Muséum national d’Histoire naturelle,* Paris 4: 287–302.

Lelièvre H, Janvier P, Goujet D. 1981. Les vertébrés dévoniens de l’Iran Central IV: arthrodires et ptyctodontes. *Géobios* 14: 677–709.

Lelièvre H, Feist R, Goujet D, Blieck A. 1987. Les vertébrés dévoniens de la Montagne Noire (sud de la France) et leur apport à la phylogénie des pachyostéomorphes (Placodermes Arthrodires). *Palaeovertebrata* 17: 1– 26.

Long JA. 1987. A new dinichthyid fish (Placodermi: Arthrodira) from the Upper Devonian ofWestern Australia, with a discussion of dinichthyid interrelationships. *Records of the Western Australian Museum* 13: 515–540.

Long JA. 1995. A new groenlandaspidid arthrodire (Pisces; Placodermi) from the Middle.

*Records of the Western Australian Museum* 17: 35-41.

Miles RS, Westoll TS. 1968. IX.—the Placoderm fish Coccosteus cuspidatus Miller ex Agassiz 743 from the middle old red sandstone of Scotland. Part I. Descriptive morphology. *Earth and environmental science transactions of the Royal Society of Edinburgh* 67(9): 373-476. [DOI 10.1017/S0080456800024078](https://doi.org/10.1017/S0080456800024078)

Miles RS. 1971. The Holonematidae (placoderm fishes), a review based on new specimens of Holonema from the Upper Devonian of Western Australia. *Philosophical Transactions of the Royal Society of London* 263: 101–234.

Miles RS. 1973. An actinolepid arthrodire from the lower Devonian Peel Sound formation,

Prince of Wales Island. *Palaeontographica Abteilung A* 109-118.

Young GC. 1981. New Early Devonian brachythoracids (placoderm fishes) from the Taemas – Wee Jasper region of New South Wales. *Alcheringa* 5: 245–271.

Stensiö EA. 1959. On the pectoral fin and shoulder girdle of the arthrodires. *Kungliga Svenska Vetenskapsakademiens Handlingar* 8:1–229.

Zhu Y-A, Zhu M, Wang, J-Q. 2015. Redescription of Yinostius major (Arthrodira: Heterostiidae) from the Lower Devonian of China, and the interrelationships of Brachythoraci. *Zoological Journal of the Linnean Society* 176(4):806-34. [DOI doi.org/10.1111/zoj.12356](https://doi.org/10.1111/zoj.12356)
